# Relationship between Advanced Glycation End Products and Plaque Progression in Patients with Acute Coronary Syndrome: The JAPAN-ACS Sub-study

**DOI:** 10.1186/1475-2840-12-5

**Published:** 2013-01-07

**Authors:** Yoshifumi Fukushima, Hiroyuki Daida, Takeshi Morimoto, Takatoshi Kasai, Katsumi Miyauchi, Sho-ichi Yamagishi, Masayoshi Takeuchi, Takafumi Hiro, Takeshi Kimura, Yoshihisa Nakagawa, Masakazu Yamagishi, Yukio Ozaki, Masunori Matsuzaki

**Affiliations:** 1 Department of Cardiology, Juntendo University School of Medicine, Tokyo, Japan; 2 Center for General Internal Medicine and Emergency Care, Kinki University School of Medicine, Osakasayama, Japan; 3 Department of Pathophysiology and Therapeutics of Diabetic Vascular Complications, Kurume University School of Medicine, Kurume, Japan; 4 Department of Advanced Medicine, Medical Research Institute, Kanazawa Medical University, Uchinada, Japan; 5 Division of Cardiology, Department of Medicine, Nihon University School of Medicine, Tokyo, Japan; 6 Department of Cardiovascular Medicine, Kyoto University Graduate School of Medicine, Kyoto, Japan; 7 Department of Cardiology, Tenri Hospital, Nara, Japan; 8 Division of Cardiovascular Medicine, Kanazawa University Graduate School of Medicine, Kanazawa, Japan; 9 Division of Cardiology, Fujita Health University, Toyoake, Japan; 10 Yamaguchi University, Ube, Japan

**Keywords:** Advanced glycation end products, Acute coronary syndrome, Intravascular ultrasound, Plaque, Statins

## Abstract

**Background:**

The Japan Assessment of Pitavastatin and Atorvastatin in Acute Coronary Syndrome (JAPAN-ACS) trial demonstrated that early aggressive statin therapy in patients with ACS significantly reduces plaque volume (PV). Advanced glycation end products (AGEs) and the receptors of AGEs (RAGE) may lead to angiopathy in diabetes mellitus (DM) and may affect on the development of coronary PV. The present sub-study of JAPAN-ACS investigates the association between AGEs and RAGE, and PV.

**Methods:**

Intravascular ultrasound (IVUS)-guided percutaneous coronary intervention (PCI) was undertaken, followed by the initiation of statin treatment (either 4 mg/day of pitavastatin or 20 mg/day of atorvastatin), in patients with ACS. In the 208 JAPAN-ACS subjects, PV using IVUS in non-culprit segment > 5 mm proximal or distal to the culprit lesion and, serum levels of AGEs and soluble RAGE (sRAGE) were measured at baseline and 8–12 months after PCI.

**Results:**

At baseline, no differences in the levels of either AGEs or sRAGE were found between patients with DM and those without DM. The levels of AGEs decreased significantly with statin therapy from 8.6 ± 2.2 to 8.0 ± 2.1 U/ml (p < 0.001), whereas the levels of sRAGE did not change. There were no significant correlations between changes in PV and the changes in levels of AGEs as well as sRAGE. However, high baseline AGEs levels were significantly associated with plaque progression (odds ratio, 1.21; 95% confidence interval, 1.01 - 1.48; p = 0.044) even after adjusting for DM in multivariate logistic regression models.

**Conclusions:**

High baseline AGEs levels were associated with plaque progression in the JAPAN-ACS trial. This relationship was independent of DM. These findings suggest AGEs may be related to long-term glucose control and other oxidative stresses in ACS.

**Trial registration:**

NCT00242944

## Background

Recent advances in plaque imaging have enabled the quantitative measurement of plaque volume (PV) and their characteristics[1,2]. Several clinical trials have observed significant plaque regression after treatment with HMG CoA-reductase inhibitors (statins) using intravascular ultrasound (IVUS) [3-6].

Using IVUS, the Japan Assessment of Pitavastatin and Atorvastatin in Acute Coronary Syndrome (JAPAN–ACS) trial has demonstrated that early aggressive statin therapy (either pitavastatin or atorvastatin) in patients with acute coronary syndrome (ACS) significantly reduces PV of non-culprit coronary lesions during the first 8–12 months after ACS [7]. In this trial, the regression of PV induced by statin therapy was smaller in diabetic patients compared with non-diabetic patients although the degree of low density lipoprotein cholesterol (LDL–C) reduction was similar between the diabetic and non-diabetic patients [8]. Recent observations from a coronary atherosclerosis study measuring the effects of rosuvastatin using intravascular ultrasound in Japanese subjects (COSMOS) indicated that poor baseline glycemic control were associated with the plaque progression [9]. Similar observations were consistently reported from recent IVUS trials that indicated that diabetic plaque has specific characteristics and/or that diabetic patients may have specific pathophysiology that attenuates the effect of statins on atherosclerotic plaque [10-13].

Several causal pathways have been proposed regarding atherogenesis in diabetes mellitus including enhanced production of reactive oxygen species, activation of protein kinase C, stimulation of the polyol pathway, and postprandial hyperglycemia[14]. Advanced glycation end products (AGEs) and receptors of AGEs (RAGE) may also contribute to the development of micro- and macroangiopathy in diabetes mellitus which may affect coronary plaque progression as well as vascular remodeling [15-19]. The present sub-study of JAPAN-ACS investigates the role of serum levels of AGEs and soluble RAGE (sRAGE) on plaque progression or regression, as observed by IVUS in JAPAN–ACS.

## Methods

### Study design

The design of the JAPAN-ACS study has been previously published [7-9]. In brief, the JAPAN–ACS study, which was conducted with the participation of 33 centers (Additional file 1), was a prospective, randomized, open-label, parallel-group study with blind endpoint evaluation. It examined the possible effect of 8–12 months of treatment with a statin on coronary plaque regression, measured using IVUS, at nonculprit lesions of the culprit vessel in patients with ACS. ACS patients satisfying all inclusion criteria were selected for participation in the study after they underwent successful, intravascular ultrasound-guided percutaneous coronary intervention (PCI). The patients were randomized within 72 hours of PCI to receive either 4 mg of pitavastatin or 20 mg of atorvastatin daily. IVUS examination was performed at baseline and was repeated 8–12 months after the start of statin treatment. Because there was no significant difference in percent change in PV between the two statin groups, the following analyses were evaluated in the full analysis set of patients. This JAPAN–ACS subgroup analysis investigated the relationships between the serum levels of AGEs or sRAGE and change of PV. The JAPAN–ACS study and related sub-studies were conducted according to the Declaration of Helsinki and with the approval of the institutional review boards of all the 33 participating institutions. Written informed consent to participate was obtained from all patients enrolled in the study.

### Biochemical analysis

Blood examinations for levels of lipid and fasting blood glucose levels, hemoglobin A1c (HbA1c), and serum levels of AGEs and sRAGE were performed at baseline and at follow-up after 8–12 months. Serum levels of the lipid profile and blood examinations as well as the levels of AGEs and sRAGE were measured at the centralized laboratory (Hokuriku University, Kanazawa and Kurume University, Kurume). Measurement of the serum levels of AGEs was performed using a competitive ELISA [20]. In this study, 1 unit (U) corresponds to 1 μg of glyceraldehyde-derived AGE-bovine serum albumin standard. sRAGE was determined using a commercially available ELISA kit (Quantikine®, R&D systems, Minneapolis, MN, USA) according to the supplier’s recommendation. The HbA1c (%) was estimated as a National Glycohemoglobin Standardization Program (NGSP) equivalent value (%) calculated using the formula HbA1c (%) = 1.02 × HbA1c (JDS; %) + 0.25% [21]. Low density lipoprotein- cholesterol (LDL-C) was calculated using the Friedewald formula [22].

### Intravascular ultrasound examination

After IVUS-guided PCI of the culprit lesion in patients with ACS, IVUS examination was performed in the longest and least angulated segment of the culprit vessel, if it met the previously described inclusion criteria. Following intracoronary administration of 200 μg of nitroglycerin, a 40-MHz, 2.6F IVUS catheter (Atlantis SR Pro2, Boston Scientific, Natik, MA, USA) was advanced into the culprit vessel, and the transducer was positioned as far distally as could be safely reached. This procedure was designed to select the longest-possible vessel segment for analysis. A motorized pullback device withdrew the transducer at a speed of 0.5 mm/s. The consoles used were ClearView or Galaxy 2 systems (Boston Scientific, Natik, MA, USA). The same imaging system with the same type of IVUS catheter was used for both the baseline and follow-up evaluations.

Two independent experienced investigators, who were unaware of the patient group allocation, performed the quantitative IVUS analysis at the central core -laboratory (Yamaguchi University, Ube) as described previously [7]. In brief, the target segment was determined as a non-culprit coronary artery segment >5 mm proximal or distal to the culprit lesion with a reproducible index, usually abranch site, on the PCI vessel. Spotty calcification, side vein, and distances from side branch, orifice, left anterior descending-left circumflex branch bifurcation, and stent edge also were referred. Then, every 6 ^th^ image (0.1-mm apart) was manually traced on a commercially available software for IVUS measurement (echoPlaque2, INDEC systems Inc., Santa Clara, CA, USA). This software automatically interpolated the tracings of five cross-sections in between the two manually traced images. Therefore, the volume was calculated from segments that were 0.017-mm thick. The IVUS measurements were performed according to the standards of the American College of Cardiology and the European Society of Cardiology [1]. The percent change in the PV during the observation period was calculated using following formula: { [ PV (follow-up) - PV (baseline) ] / PV (baseline) } × 100. Regression of coronary PV was defined as percent change in PV <0% and progression of coronary PV was defined as percent change in PV ≥0%.

### Statistical analysis

Continuous variables are presented as the mean ± SD or median (interquartile range; IQR) according to their distribution. Categorical variables are expressed as numbers and percentages. We used Pearson correlations to assess the relationships between AGEs, sRAGE, fasting blood glucose, and HbA1c. Within-group and between-group comparisons were carried out using paired or unpaired Student’s *t-*tests for normally distributed data The Wilcoxon signed rank test or Wilcoxon rank sum test was used for non-normally distributed data. The chi-square test or Fisher’s exact test was used for categorical variables.

To test whether the levels of AGEs or sRAGE were significantly associated with plaque progression, we developed logistic regression models with plaque progression as the independent variable. When either AGEs or sRAGE were associated with plaque progression in the univariate model, we developed the multivariate model. We included in the multivariate model the following variables which were significantly associated with the degree of coronary PV change in the previous report of the JAPAN–ACS sub-study [8], diabetes mellitus, baseline PV and baseline remnant like lipoprotein cholesterol (RLP-C) in addition to AGEs or sRAGE. The continuous variables were treated as continuous in the logistic models, and effects were expressed by odds ratio (OR) and 95% confidence intervals (CI) for 1 unit increments against plaque progression.

The relationships between percent changes of PV and those of serum levels AGEs as well as sRAGE. A value of p < 0.05 was considered significant. All statistical analyses were performed using the SPSS, Ver.11.0 (SPSS Inc, Chicago, Illinois).

## Results

### Baseline AGEs and sRAGE levels and their changes

A total of 208 ACS patients, for whom frozen serum samples were available to measure levels of AGEs and sRAGE, were enrolled in this study. The baseline serum levels of AGEs and sRAGE in these patients were 8.6 ± 2.2 U/ml and 692 (493 – 1037) pg/ml, respectively. The relationship between baseline characteristics and AGEs or sRAGE level is shown in Table 1. The serum level of AGEs was higher in smokers than in non-smokers, and it tended to be higher in men than in women. No differences in either AGE or sRAGE levels were observed between diabetic patients and nondiabetic patients (Table 1).

**Table 1:** Baseline AGEs and sRAGE levels

**Background**		**N = 208**	AGEs^*^(U/mL)	**P-value**	sRAGE^*^(pg/mL)	**P-value**
Statin	Atorvastatin	98	8.56 ± 2.35	0.99	736 (494–1099)	0.6
	Pitavastatin	110	8.56 ± 2.04		681 (492–1016)	
Gender	Women	42	8.00 ± 2.22	0.06	704 (413–1104)	0.8
	Men	166	8.71 ± 2.17		692 (494–1004)	
Diabetes mellitus	No	146	8.44 ± 2.14	0.2	692 (481–1015)	0.2
	Yes	62	8.86 ± 2.28		755 (552–1096)	
Hypertension	No	81	8.46 ± 2.22	0.6	684 (489–1022)	0.8
	Yes	127	8.63 ± 2.18		718 (494–1095)	
Smoking	No	112	8.25 ± 2.14	0.028	692 (541–1050)	0.5
	Yes	96	8.92 ± 2.20		707 (453–1037)	
ST elevation	No	74	8.63 ± 2.10	0.7	720 (537–949)	0.8
	Yes	134	8.53 ± 2.24		683 (472–1080)	

*Data of AGEs were expressed as mean ± SD and those of sRAGE were median (interquartile range). AGEs, Advanced glycation end products; sRAGE, serum Receptor of AGE.

In addition, there was no correlation between AGEs or sRAGE levels and either fasting blood glucose levels (r = 0.08, p = 0.3 for AGEs, r = 0.07, p = 0.3 for sRAGE), or HbA1c levels (r = 0.09, p = 0.2 for AGEs, r = 0.09, p = 0.2 for sRAGE).

**Figure 1:**
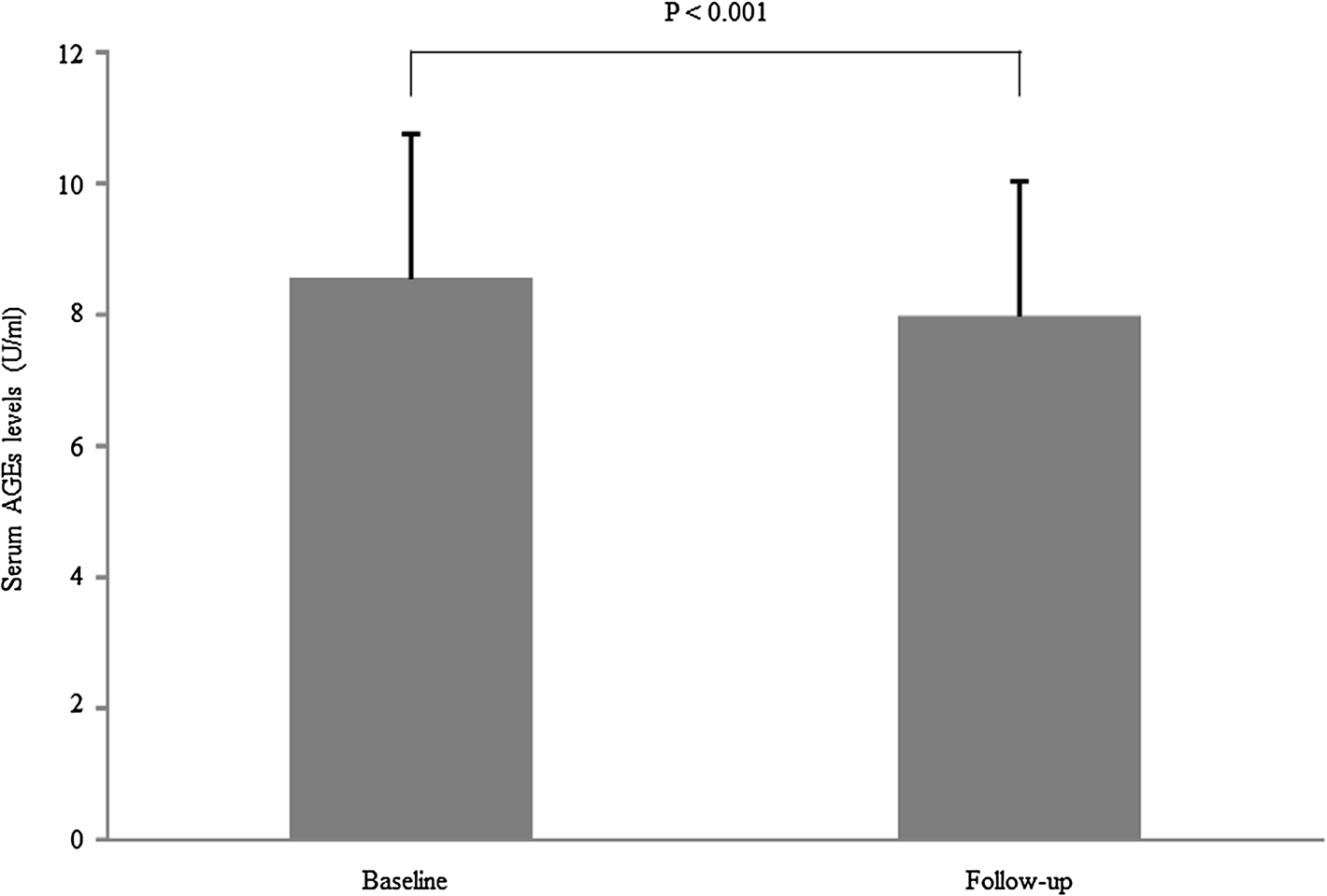
**Change in serum AGEs levels (n=208).** Data of AGEs were expressed as mean ± SD. Serum AGE levels decrease significantly from baseline to follow-up (8.6 ± 2.2 to 8.0 ± 2.1 U/ml, p < 0.001). AGEs, Advanced glycation end products.

Levels of AGEs had decreased significantly (8.6 ± 2.2 to 8.0 ± 2.1 U/ml, p < 0.001) upon follow-up (Figure 1), whereas sRAGE levels had not decreased significantly [692 (493 – 1037) to 736 (532 – 990) pg/ml, p = 0.4]. The AGEs level in the pitavastatin group (n = 110) had decreased significantly (8.6 ± 2.0 to 7.7 ± 1.8 U/ml, p < 0.001), whereas in the atorvastatin group (n = 98) it had not (8.6 ± 2.4 to 8.3 ± 2.3 U/ml, p = 0.051).

### Comparison between patients with plaque regression and those with plaque progression

Regression of plaque had occurred in 180 patients and the remaining 28 patients had showed progression. Comparisons between the two groups in clinical characteristics and laboratory results are shown in Tables 2 and 3, respectively. Patients with plaque regression were less likely to have diabetes mellitus. In addition, patients with plaque regression had significantly lower baseline levels of AGEs than those with progression (8.4 ± 2.2 vs. 9.4 ± 1.9 U/ml, p = 0.032), whereas no significant differences were observed in the baseline level of sRAGE.

**Table 2:** Comparison in clinical characteristics between patients with plaque regression and progression

	**Regression**	**Progression**	**P-value**
	**N=180**	**N=28**	
Age (yrs)	62.4 ± 10.6	65.8 ± 13.6	0.1
Male	142 (78.9)	24 (85.7)	0.4
BMI (kg/m^2^)	24.3 ± 3.5	23.6 ± 2.5	0.2
Diabetes mellitus	48 (26.7)	14 (50.0)	0.016
Hypertension	107 (59.4)	20 (71.4)	0.2
Smoking	85 (47.2)	11 (39.3)	0.4
History of prior PCI	12 (6.7)	1 (3.6)	0.5
Type of ACS			0.3
STEMI	113 (62.8)	21 (75.0)	
NSTEMI	26 (14.4)	4 (14.3)	
UAP	41 (22.8)	3 (10.7)	
Culprit Vessel			0.1
RCA	56 (31.1)	14 (50.0)	
LAD	99 (55.0)	13 (46.4)	
LCX	24 (13.3)	1 (3.6)	
LMT	1 (0.6)	0 (0)	
Type of stent			0.6
BMS	120 (66.7)	19 (67.9)	
DES	57 (31.7)	9 (32.1)	
Other than Stent (POBA)	3 (1.7)	0 (0)	
Analysis Segment			0.6
Proximal to target site	124 (68.9)	18 (64.3)	
Distal to target site	56 (31.1)	10 (35.7)	
Use of pitavastatin	96 (53.3)	14 (50.0)	0.7

BMI, body mass index; PCI, percutaneous coronary intervention; ACS, acute coronary syndrome; STEMI, ST-elevation myocardial infarction; NSTEMI, non-ST-elevation myocardial infarction; UAP, unstable angina pectoris; RCA, right coronary artery; LAD, left anterior descending artery; LCX, left circumflex branch; LMT, left main trunk; BMS, bare-metal stent; DES, drug-eluting stent; POBA, plain old balloon angioplasty.

Data are expressed in numbers (percentage), unless otherwise specified. Age and BMI are presented as the mean ± SD. * < 0.05 vs. patients with regression.

**Table 3:** Comparison in laboratory results between patients with plaque regression and progression

	**Regression**	**Progression**	**P-value**
	**N=180**	**N=28**	
WBC (cells/μl)	9323 ± 3443	9635 ± 1878	0.5
Max CK (IU/L)	1852 ± 1993	1717 ± 1629	0.7
Hb (g/dL)	14.1 ± 1.6	13.7 ± 1.9	0.3
AST (IU/L)	80.2 ± 102.8	79.2 ± 86.9	0.96
ALT (IU/L)	33.9 ± 22.5	35.2 ± 22.4	0.8
γ-GTP (IU/L)	45.7 ± 52.3	57.2 ± 68.3	0.4
Total bilirubin (mg/dL)	0.8 ± 0.3	0.8 ± 0.3	0.2
BUN (mg/dL)	15.3 ± 4.9	15.4 ± 5.2	0.9
Creatinine (mg/dL)	0.81 ± 0.24	0.83 ± 0.19	0.7
Uric acid (mg/dL)	5.4 ± 1.6	5.1 ± 1.7	0.4
TC (mg/dL)	196.1 ± 36.5	196.3 ± 35.4	0.98
LDL-C (mg/dL)	132.1 ± 33.3	131.4 ± 34.0	0.9
TG (mg/dL)	115.2 ± 53.0	115.1 ± 24.0	0.99
HDL-C (mg/dL)	44.0 ± 10.3	45.7 ± 8.8	0.4
RLP-C (mg/dL)	4.2 ± 2.3	4.0 ± 2.3	0.6
hs-CRP(mg/L)	32.8 ± 39.0	48.8 ± 43.9	0.08
FBS (mg/dL)	153.6 ± 61.8	156.9 ± 63.8	0.8
HbA1c (%)	6.3 ± 1.2	6.9 ± 1.7	0.023
AGEs (U/mL)	8.4 ± 2.2	9.4 ± 1.9	0.032
sRAGE (pg/mL)	683 (496–1037)	723 (483–1081)	0.9

WBC, white blood cell counts; CK, creatine kinase; Hb, hemoglobin; AST, aspartate aminotransferase; ALT, alanine aminotransferase; γ-GTP, γ-guanosine 5’-triphosphate; BUN, blood urea nitrogen; TC, total cholesterol; LDL-C, low density lipoprotein-cholesterol; TG, triglyceride; HDL-C, high density lipoprotein cholesterol; hs-CRP, high-sensitivity C-reactive protein; FBS, fasting blood sugar; HbA1c, haemoglobin A1c; AGEs, Advanced glycation end products; sRAGE, serum Receptor of AGE.

Data were expressed as the mean ± SD or median (IQR).

### The relationship between serum levels of AGEs and plaque progression

There was a significant relationship between the baseline level of AGEs and the progression of PV (OR 1.23, 95% CI 1.02 –1.50, p = 0.030), whereas there was no significant relationship between the baseline level of sRAGE and the progression of PV in univariate models (OR 1.00, 95% CI 0.999 – 1.000, p = 0.5). Multivariate logistic regression model with baseline AGEs, diabetes mellitus, baseline PV and baseline RLP-C showed that baseline level of AGEs was significantly associated with plaque progression (OR 1.21, 95% CI 1.01 – 1.48, p = 0.044) (Table 4).

**Table 4:** Variables associated with plaque progression

**Parameter**	**OR (95% CI)**	**P-value**
Baseline AGEs	1.21 (1.01 - 1.48)	0.044
Diabetes mellitus	2.62 (1.14 - 6.05)	0.024
Baseline plaque volume	1.00 (0.99 - 1.02)	0.6
Baseline RLP-C	0.91 (0.72 - 1.10)	0.4

The continuous variables were treated as continuous in the logistic models, and effects were expressed by odds ratio for 1 unit increments.

AGEs, Advanced glycation end products; RLP-C, remnant like particle- cholesterol.

### Correlation between plaque volume and the change in serum levels of AGEs or sRAGE

There was no significant correlation between the percent change in PV and the change in serum levels of AGEs (r = − 0.07, p = 0.3). The percent change in sRAGE levels during the study period was also not significantly correlated with the percent change in PV (r = − 0.02, p = 0.8).

## Discussion

The primary results of JAPAN–ACS indicated that diabetes mellitus attenuated the effect of statins in terms of plaque regression. The present sub-study of JAPAN-ACS investigates the association between plaque progression/regression and the serum levels of AGEs as well as sRAGE. The baseline AGEs level was significantly higher in patients with plaque progression (8.4 ± 2.2 vs. 9.4 ± 1.9U/ml), although this association was independent of diabetes mellitus. In addition, there was no correlation between the change in AGEs levels and percent change in PV, although the AGEs levels reduced significantly during 10 months of follow-up. The sRAGE levels did not demonstrate any correlation with plaque progression or regression. Further, in the present study, although the AGEs levels were not different between patients with diabetes and those without, they were significantly higher in smokers than in nonsmokers, and they tended to be higher in men than in women.

There is accumulating evidence that AGEs and RAGE are implicated in the pathogenesis of various devastating disorders such as diabetic vascular complications, cardiovascular disease, Alzheimer’s disease, cancer growth and metastasis, insulin resistance and non-alcoholic steatohepatitis [15,16,23-25]. Harmful effects of AGEs were predominantly observed in large vessels [26-28]. Indeed, it was reported that AGEs decreased large vessel elasticity and elicited inflammatory and pro-thrombotic responses in the vessel wall, thereby being involved in vascular complications [26-28]. Furthermore, the expression levels of AGEs and RAGE were increased in diabetic atherosclerotic lesions [29,30] Because AGEs are generated not only from Amadori products under hyperglycemic conditions, but also from dicarbonyl compounds derived from the Maillard reaction, the auto-oxidation of reducing sugars and other metabolic pathways of glucose [15]. They include heterogeneous molecules such as carboxymethyllysine, pentosidine, and glyceraldehyde-derived AGEs [23-25]. In the present study, we measured levels of glyceraldehyde-derived AGEs, which could reflect inflammatory oxidative stress conditions and/or hyperglycaemic states [23-25]. Thus the levels of AGEs measured here may be influenced by both the presence of diabetes mellitus and its long-term control of hyperglycemia, and by additional oxidative stress conditions such as smoking status.

In this study, the baseline levels of AGEs were significantly higher in patients with plaque progression than in those without progression, although the reduction in the levels of AGEs did not correlate with the change in PV. In the multivariate logistic regression analysis, the presence of diabetes mellitus and baseline level of AGEs were significant, independent variables associated with the plaque progression. This further supports the pathological role for AGEs in accelerated atherosclerosis in humans.

In the present study, although statin therapy reduced the levels of AGEs, this reduction did not correlate with the change in PV. AGEs are hardly degraded and remain for a long time in diabetic vessels even after glycemic control and oxidative stress conditions have been improved [15]. Therefore, the phenomenon of so-called metabolic memory could be explained, in part, by AGEs [31]. In other words, the harmful effects of AGEs on plaque progression may not be easily reversed by short-term statin therapy. Former smokers remain at an increased risk for developing lung cancer and cardiovascular disease even years after they stop smoking [32]. Because this phenomenon has an interesting analogy to metabolic memory, and because tobacco smoking is a major environmental source of AGEs in humans [32], the deleterious effects of smoking on atherosclerosis could be mediated, in part, by AGEs. In this study, the levels of sRAGE did not show any relationship to plaque progression or regression. Although RAGE definitely plays an important role in atherosclerotic progression [33,34], the value of measuring sRAGE levels remains uncertain. Further study is warranted.

Although the present study indicated the possible adverse effect of AGEs on plaque regression during the statin therapy, some limitations should be considered. First, AGEs and sRAGE levels were measured in 208 frozen samples from patients who participated in JAPAN–ACS that only accounted for two-thirds of all participants. However, the lack of sample did not occur systematically, and the effects were small if present. Second, the end point of the JAPAN–ACS trial was the change in total atheroma volume, whose index may not be affected by the AGE–RAGE axis. However, given the pivotal role of the AGE–RAGE axis in experimental atherosclerosis [15,28-30], the present findings could provide some new insight into the pathophysiology of plaque progression and regression in patients with ACS. Finally, the number of patients with plaque progression was small. Thus, to avoid the overloading of multivariate model, we could not include all potential confounders. We thus used the factors associated with in the previous study to adjust the confounding as much as possible.

## Conclusion

High baseline levels of AGEs were associated with PV progression in the JAPAN–ACS trial. In addition, this relationship was independent of the presence of diabetes mellitus and HbA1c levels. Therefore, the mechanism by which AGEs affects plaque progression may not be related to the presence of diabetes mellitus itself, but to long-term cumulative hyperglycemia and/or oxidative stress conditions in patients with ACS. Further studies are needed to elucidate the role of the AGE–RAGE axis in the progression of atherosclerosis in humans.

## Competing interests

The Japan Heart Foundation funded the JAPAN-ACS study with an unrestricted grant from Kowa Pharmaceutical. Kowa pharmaceutical participated in the preparation of the study design. However, the investigators made the final decision on the study design, database maintenance, made manuscript, and submission of the article including sub-analyses.

Dr. Fukushima has no conflict of interest. Dr. Daida has received honoraria for the lectures and research grants from Kowa pharmaceutical, Pfizer and Astellas Pharma. Dr. Morimoto has received honoraria for the lectures from Kowa pharmaceutical and Pfizer, and served as consultant of data safety monitoring board for Pfizer. Dr Kasai has no conflict of interest. Dr. Miyauchi has received honoraria for the lectures from Kowa pharmaceutical, Pfizer and Astellas Pharma. Dr. S Yamagishi has received honoraria for the lectures and research grants from Kowa pharmaceutical, Pfizer and Astellas Pharma. Dr. Takeuchi has received honoraria for the lectures from Astellas Pharma, and.research grants from Kowa pharmaceutical and Astellas Pharma. Dr. Hiro has received honoraria for the lectures from Kowa pharmaceutical, Pfizer and Astellas Pharma. Dr. Kimura has received honoraria for the lectures from Kowa pharmaceutical, Pfizer and Astellas Pharma, and research grant from Kowa pharmaceutical. Dr. Nakagawa has received honoraria for the lectures from Kowa pharmaceutica, Pfizer and Astellas Pharma. Dr. M Yamagishi has received honoraria for the lectures from Kowa pharmaceutical, Pfizer and Astellas Pharma and has received research grant from Kowa pharmaceutical and Astellas Pharma. Dr. Ozaki has received honoraria for the lectures from Pfizer and Kowa pharmaceutical, and research grant from Kowa pharmaceutical. Dr. Matsuzaki has received honoraria for the lectures from Kowa pharmaceutical, Pfizer and Astellas Pharma, and research grant from Pfizer and Astellas Pharma.

## Authors' contributions

All authors conceived the study and participated in its design. TM and TK performed the statistical analysis. YF, HD and KM drafted the manuscript and interpreted rhe data. All authors have read and approved the final manuscript.

## Supplementary Material

Additional file 1 JAPAN-ACS investigators. Click here for file
