# Supplementary material for: Relationship between Advanced Glycation End Products and Plaque Progression in Patients with Acute Coronary Syndrome: The JAPAN-ACS Sub-study
Source: Cardiovasc Diabetol. 2013 Jan 7;12:5. doi: 10.1186/1475-2840-12-5 (PMC3571912; doi:10.1186/1475-2840-12-5)
Supplement: Additional file 1 — JAPAN-ACS investigators. [file 1475-2840-12-5-S1.pdf]

Additional file 1

JAPAN-ACS Investigators:

Aichi Medical University (Hiroaki Takashima, MD, Kenji Asai, MD, Yasushi Kuhara, MD); Akita Medical Center (Tadaya Sato, MD); Anjo Kosei Hospital (Masato Watarai, MD, Kenji Takamoto, MD); Ehime Prefectural Central Hospital (Hideki Okayama, MD); Fujita Health University (Yukio Ozaki, MD, Hiroyuki Naruse, MD); Fukuoka University (Keijiro Saku, MD, Shin-ichiro Miura, MD, Atsushi Iwata, MD); Gifu Prefectural General Medical Center (Toshiyuki Noda, MD, Shunichiro Warita); Hiroshima City Asa Hospital (Keigo Dote, MD, Masaya Kato, MD); Hiroshima City Hospital (Ichiro Inoue, MD, Takuji Kawagoe, MD); Japanese Redcross Nagoya First Hospital (Haruo Kamiya, MD); Juntendo University (Hiroyuki Daida, MD, Katsumi Miyauchi, MD); Juntendo University Shizuoka Hospital (Satoru Suwa, MD); Juntendo University Urayasu Hospital (Yuji Nakazato, MD, Kosei Tanimoto, MD); Kansai Rosai Hospital (Masaaki Uematsu, MD, Fusako Sera ,MD); Kokura Memorial Hospital Masashi Iwabuchi, MD, Shinichi Shirai, MD); Kurashiki Central Hospital (Kazuaki Mitsudo, MD, Yasushi Fuku, MD); Kyoto University (Takeshi Kimura, MD, Y Tomoya Tada, MD); National Cardiovascular Center (Hiroshi Nonogi, MD); National Hospital Organization Hamada Medical Center (Hiroshi Iida, MD); National Hospital Organization Kagoshima Medical Center (Shinichi Minagoe, MD, Hitoshi Nakashima, MD, Masahiro Sonoda, MD, Hideki Tanaka, MD); NTT East Corporation Sapporo Hospital (Tetsuro Kouya, MD, Noriyuki Miyamoto, MD); Okayama Redcross General Hospital (Toru Ujihira, MD); Osaka City General Hospital (Akira Itoh, MD, Kazato Ito, MD); Saiseikai Kumamoto Hospital (Koichi Nakao, MD); Sendai Kosei Hospital (Taiichiro Meguro, MD, Kaname Takizawa, MD); The University of Tokyo (Yasunobu Hirata, MD, Jiro Ando, MD);Tokuyama Central Hospital (Hiroshi Ogawa, MD, Takahiro Iwami, MD);

Toranomon Hospital (Sugao Ishiwata, MD, Yo Fujimoto, MD); Tsuchiya Genaral Hospital (Yasuhiko Hayashi, MD, Nobuo Shiode, MD, Mamoru Toyofuku, MD); Wakayama Medical University (Takashi Akasaka, MD, Hironori Kitabata, MD); Yamaguchi University (Masunori Matsuzaki, MD, Takayuki Okamura, MD); Yokkaichi Municipal Hospital (Masaaki Kanashiro, MD, Toru Aoyama, MD); Yokohama City University Medical Center (Kiyoshi Hibi, MD, Mitsuaki Endo, MD).
